# Towards doubling fibre yield for cotton in the semiarid agricultural area by increasing tolerance to drought, heat and salinity simultaneously

**DOI:** 10.1111/pbi.13476

**Published:** 2020-09-29

**Authors:** Nardana Esmaeili, Yifan Cai, Feiyu Tang, Xunlu Zhu, Jennifer Smith, Neelam Mishra, Eric Hequet, Glen Ritchie, Don Jones, Guoxin Shen, Paxton Payton, Hong Zhang

**Affiliations:** ^1^ Department of Biological Sciences Texas Tech University Lubbock TX USA; ^2^ College of Agronomy Jiangxi Agricultural University Nanchang China; ^3^ St. Joseph's College Autonomous Bengaluru Karnataka India; ^4^ Department of Plant and Soil Science Texas Tech University Lubbock TX USA; ^5^ Cotton Incorporated Cary NC USA; ^6^ Zhejiang Academy of Agricultural Sciences Hangzhou China; ^7^ USDA‐ARS Cropping Systems Research Laboratory Lubbock TX USA

**Keywords:** *AVP1*, co‐overexpression, drought stress, heat stress, *OsSIZ1*, salinity, transgenic cotton

## Abstract

Abiotic stresses such as extreme temperatures, water‐deficit and salinity negatively affect plant growth and development, and cause significant yield losses. It was previously shown that co‐overexpression of the Arabidopsis vacuolar pyrophosphatase gene *AVP1* and the rice SUMO E3 ligase gene *OsSIZ1* in Arabidopsis significantly increased tolerance to multiple abiotic stresses and led to increased seed yield for plants grown under single or multiple abiotic stress conditions. It was hypothesized that there might be synergistic effects between *AVP1* overexpression and *OsSIZ1* overexpression, which could lead to substantially increased yields if these two genes are co‐overexpressed in real crops. To test this hypothesis, *AVP1* and *OsSIZ1* were co‐overexpressed in cotton, and the impact of *OsSIZ1/AVP1* co‐overexpression on cotton's performance under normal growth and multiple stress conditions were analysed. It was found that *OsSIZ1/AVP1* co‐overexpressing plants performed significantly better than *AVP1*‐overexpressing, *OsSIZ1*‐overexpressing and wild‐type cotton plants under single, as well as under multiple stress conditions in laboratory and field conditions. Two field studies showed that *OsSIZ1/AVP1* co‐overexpressing plants produced 133% and 81% more fibre than wild‐type cotton in the dryland conditions of West Texas. This research illustrates that co‐overexpression of *AVP1* and *OsSIZ1* is a viable strategy for engineering abiotic stress‐tolerant crops and could substantially improve crop yields in low input or marginal environments, providing a solution for food security for countries in arid and semiarid regions of the world.

## Introduction

Abiotic stresses refer to unfavourable growth conditions for plants that include high and low temperatures, water‐deficit, salinity, nutrient starvation, and high light intensity. Abiotic stresses are responsible for substantial yield losses annually for most crops. The upland cotton (*Gossypium hirsutum* L.) is an economically important crop for textile industry, providing 35% of the total fibre used worldwide (Abdelraheem *et al*., 2019). India, USA, China, Brazil and Pakistan produced most cotton fibres in the world (Abdelraheem *et al*., 2019). Except for Brazil, cotton production in the other four countries is mainly in regions where irrigation water is severely limited. In 2015, the United States Department of Agriculture predicted that a future decline in cotton production would likely occur because of drought stress. Indeed, cotton industry has been severely affected by drought and heat stresses, leading to a loss of fibre yield by 34% recently (Ullah *et al*., 2017).

As the world population increases, the demand for food, fresh water, fibre and energy is rising. Yet climate is changing steadily, which leads to more severe environmental stresses that can be detrimental to agricultural production. In order to meet the need for food and fibre in the coming decades, it is imperative to increase crop yield by 50% or more in the near future (Nakashima *et al*., 2014; Shaar‐Moshe *et al*., 2017). Therefore, crop production must be tailored, and the development of stress‐tolerant crops is required to sustain world agricultural production.

Several strategies were used to manage abiotic stresses in agricultural production to maintain crop yield under environmental stress conditions. Transgenic technology is one of the approaches that has been used for more than two decades towards generating stress‐resistant crops. The Arabidopsis vacuolar proton pyrophosphatase 1 (AVP1) is one of the three proton pumps in plants that generates a proton gradient across vacuolar membranes (Gaxiola *et al*., 2001). Therefore, the function of AVP1 is to maintain cell turgor and plant rigidity via generating H^+^ gradient across vacuolar membrane by pumping H^+^ into vacuole, which then can be used by antiporters on vacuolar membrane such as NHX1 to sequester Na^+^ into vacuole in exchange for H^+^, reducing sodium toxicity in cytoplasm, leading to increased salt tolerance. Furthermore, AVP1 stimulates auxin polar transport, which promotes root development, making plants absorb water more efficiently. In contrast, *avp1* null mutants display impaired root and shoot development with very low auxin transport (Li *et al*., 2005). Improved drought and salt tolerance were achieved by overexpression of *AVP1* in Arabidopsis (Gaxiola *et al*., 2001; Li *et al*., 2005), cotton (Pasapula *et al*., 2011) and peanut (Qin *et al*., 2013). Also, overexpression of *AVP1* in plants enhances rhizosphere acidification, which helps nutrient uptake under drought and salinity conditions (Yang *et al*., 2007).

The SUMO E3 ligase 1 (SIZ1) is an enzyme that mediates protein sumoylation in plants. Sumoylation is a post‐translational modification mechanism that modifies proteins through conjugation of SUMO (small ubiquitin‐like modifier) to target proteins (Li *et al*., 2013). Sumoylation plays important roles in plant response to biotic and abiotic stresses including drought (Catala *et al*., 2007), heat (Li *et al*., 2013; Yoo *et al*., 2006), cold (Miura *et al*., 2007) and salt (Conti *et al*., 2008; Miura and Nozawa, 2014). To date, a wide range of biological functions has been identified in which the SIZ1‐mediated sumoylation plays essential roles. According to early reports, ABA response is negatively regulated by the sumoylation process, particularly during seed germination and primary root growth (Lois *et al*., 2003; Miura and Hasegawa, 2010; Miura *et al*., 2009). The Arabidopsis SIZ1 is also involved in phosphate starvation and nitrogen assimilation (Miura *et al*., 2005; Park *et al*., 2011; Wang *et al*., 2015). The role of SIZ1‐dependant sumoylation in sugar signalling and metabolism in plant growth and development was discovered (Castro *et al*., 2015). A previous study showed that *siz1* mutants display abnormal root growth in glucose‐supplemented media, including the formation of abnormal root hairs, implicating that mutation in *SIZ1* causes increased root sensitivity to glucose, and that SIZ1 is a positive regulator of root growth and development in the presence of glucose (Castro *et al*., 2015). Overexpression of the rice SUMO E3 ligase gene *OsSIZ1* in cotton increased fibre yield under combined drought and heat stresses as well as in field conditions (Mishra *et al*., 2017).

It is well known that abiotic stresses often occur in combination in nature, which could cause significantly more damages to plants and reduce crop productivity far more severely. Therefore, engineering crops that can maintain high yield in marginal environments would be a major break‐through in agricultural production. Recently our proof‐of‐concept study showed that co‐overexpression of *AVP1* and *OsSIZ1* in Arabidopsis improves multi‐stress tolerance substantially, and transgenic plants produced a significantly higher yield under severe stress conditions (Esmaeili *et al*., 2019). In this study, we co‐overexpressed *OsSIZ1* and *AVP1* in cotton, and we showed that *OsSIZ1/AVP1* co‐overexpressing cotton plants demonstrated a far more superior performance than all other control plants under all abiotic stress conditions tested. Furthermore, we explored the underlying mechanism of the significantly improved abiotic stress tolerance of *OsSIZ1/AVP1* co‐overexpressing plants via transcriptome analysis of transgenic and non‐transgenic cotton plants grown under field stress conditions.

## Results

### Creation and molecular analysis of *OsSIZ1/AVP1* co‐overexpressing cotton plants

The Arabidopsis vacuolar H^+^‐pyrophosphatase gene *AVP1* and the rice SUMO E3 ligase gene *OsSIZ1* were fused to Cauliflower mosaic virus 35S promoter and a maize ubiquitin promoter, respectively, with the nopaline synthase terminator sequence used in both expression cassettes. Then, these two expression cassettes were inserted into the T‐DNA region of the pBI121‐based binary vector to form the transforming vector (Esmaeili *et al*., 2019), which was used to transform wild‐type (WT) cotton. A total of 27 independent transgenic cotton plants were generated via Agrobacterium‐mediated transformation method (Bayley *et al*., 1992). Among these putative transgenic lines, four independent lines co‐overexpressing *OsSIZ1* and *AVP1* (OA) were selected for further analyses. One *AVP1*‐overexpressing cotton line (A) and one *OsSIZ1*‐overexpressing cotton line (O) from our previous studies (Mishra *et al*., 2017; Pasapula *et al*., 2011) were used as reference lines. The segregated non‐transgenic line (SNT) of an OA plant and WT cotton plant were used as negative controls in this study.

**Figure 1:**
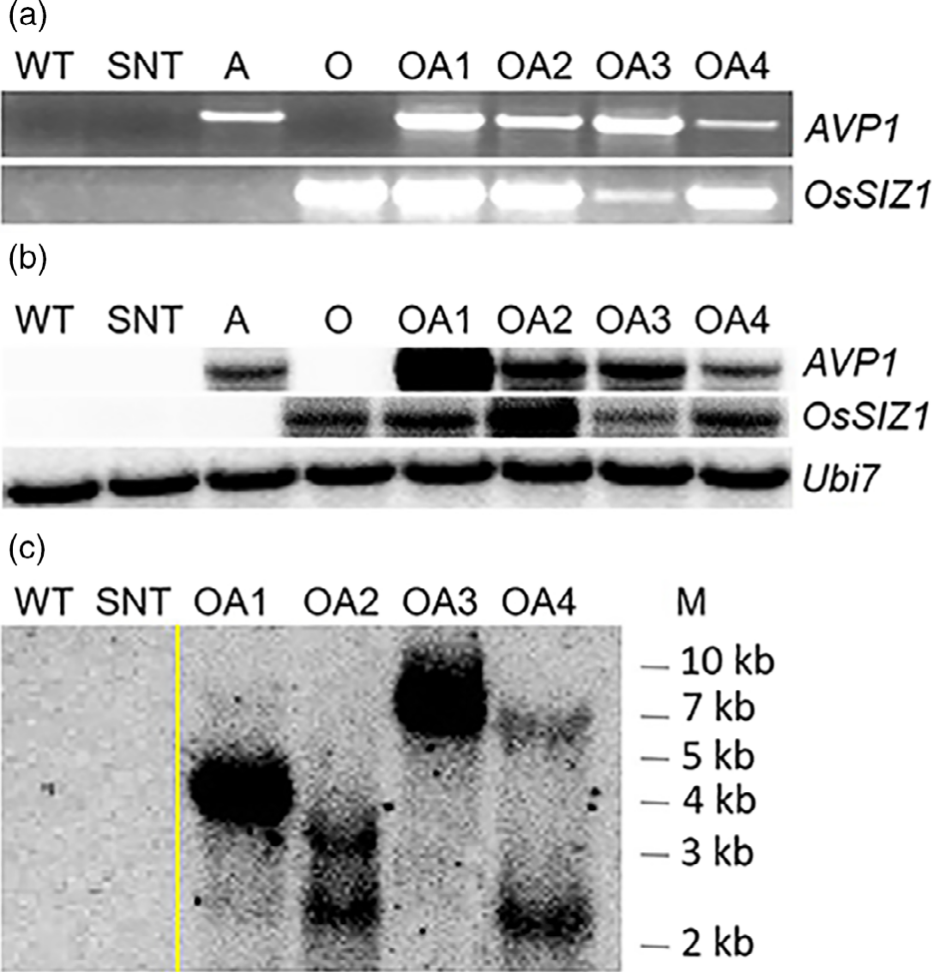
Molecular analysis of *OsSIZ1/AVP1* co‐overexpressing cotton plants. (a) PCR analysis of control and *OsSIZ1/AVP1* co‐overexpressing cotton plants. The gene fragments amplified are labelled on the right. (b) RNA blot analysis of control and *OsSIZ1/AVP1* co‐overexpressing cotton plants. The genes used as probes are listed on the right. (c) DNA blot analysis of control and *OsSIZ1/AVP1* co‐overexpressing cotton plants. Molecular weight markers are on the right. WT, wild‐type plant; SNT, segregated non‐transgenic plant; A, *AVP1*‐overexpressing plant; O, *OsSIZ1*‐overexpressing plant; OA1 to OA4, four independent *OsSIZ1*/*AVP1* co‐overexpressing plants.

Total DNAs were extracted from WT, SNT, A, O and OA plants, then used for PCR analysis using gene‐specific primers to confirm the presence of corresponding transgenes (Figure 1a). The RNA blot analysis showed that all four OA plants expressed transcripts for both transgenes, whereas A and O plants expressed transcript for *AVP1* and *OsSIZ1*, respectively (Figure 1b). As expected, no *AVP1* or *OsSIZ1* transcripts were found in WT and SNT plants. DNA blot analysis showed that OA1 and OA3 were likely single T‐DNA insertion plants, and OA2 and OA4 were likely due to two T‐DNA insertion events. WT and SNT plants did not contain transgenes *AVP1* or *OsSIZ1* (Figure 1c).

### *OsSIZ1/AVP1* co‐overexpressing plants perform the best under combined drought and salt stresses and produced the highest fibre yield

**Figure 2:**
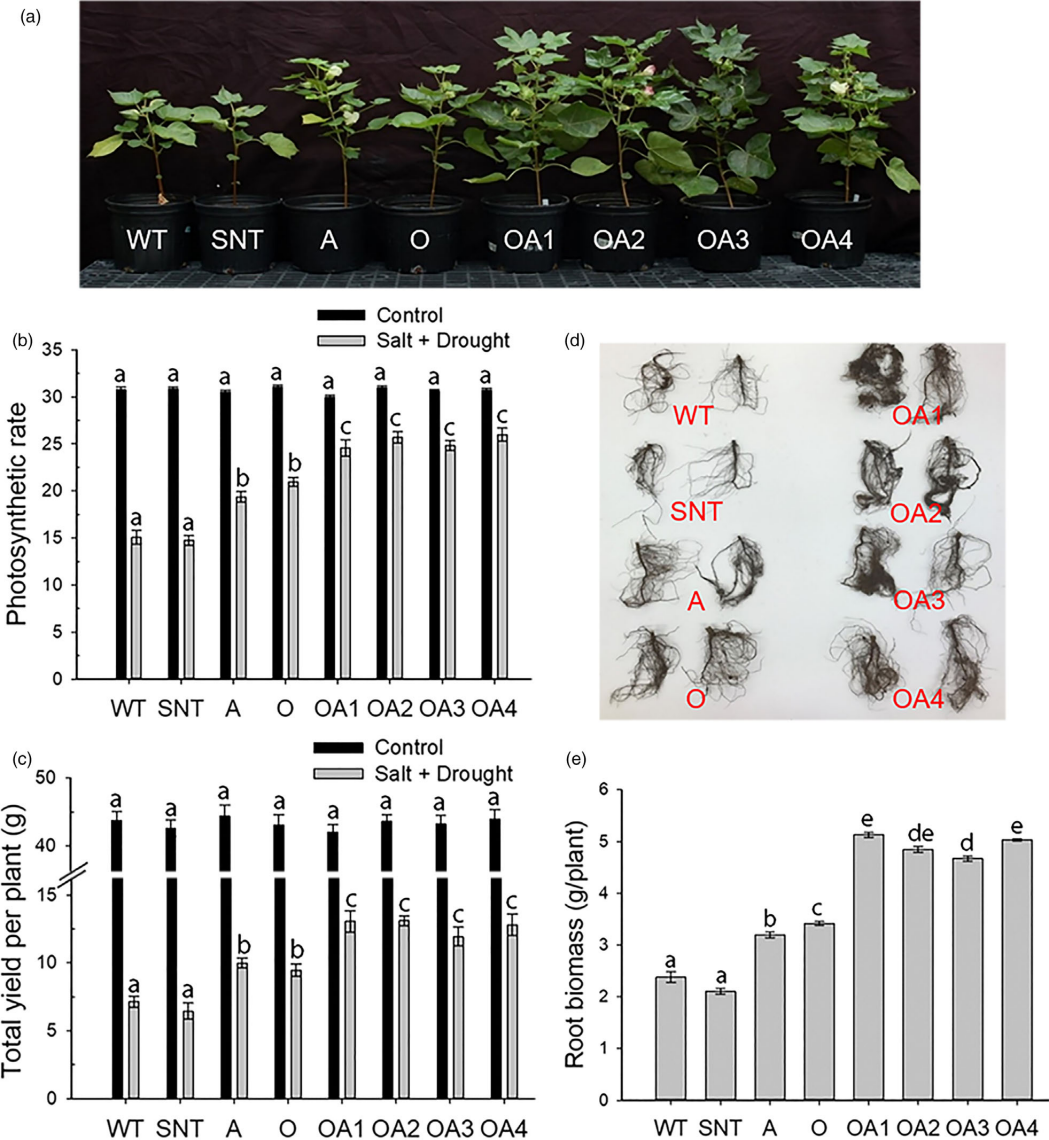
The effects of combined drought and salt stresses on control and *OsSIZ1/AVP1* co‐overexpressing cotton plants. (a) Phenotypes of cotton plants six weeks after combined drought and salt treatment. (b) Photosynthetic rates of cotton plants under normal irrigation (black bar) and combined drought and salt stresses (grey bar). (c) Total seed fibre yields under normal irrigation (black bar) and combined drought and salt stresses (grey bar). Results are the means ± SE (*n* = 9). (d) Root phenotypes of cotton plants under combined drought and salt stresses. (e) Root biomass analysis of cotton plants under combined drought and salt stresses. Results are the means ± SE (*n* = 5). WT, wild‐type plant; SNT, segregated non‐transgenic plant; A, *AVP1*‐overexpressing plant; O, *OsSIZ1*‐overexpressing plant; OA1 to OA4, four independent *OsSIZ1/AVP1* co‐overexpressing plants. Samples denoted by different letters are significantly different (*P* < 0.05, Tukey correction).

Combined drought and salt stresses are one of the worst conditions for crop growth in arid and semiarid regions due to lack of precipitation and accumulation of fertilizers in soils. Before the initiation of stress treatment, no phenotypic differences were observed among 4‐week‐old cotton plants of different genotypes (Fig. S1A), but after the treatment of combined stresses for two months, OA plants displayed the best phenotype in comparing to WT, SNT, A and O plants (Figure 2a). Photosynthetic rates of cotton plants under normal growth condition as well as under combined drought and salt stresses were measured. Although no significant differences were observed among different genotypes under a normal growth condition, OA plants displayed the highest photosynthetic rates when compared with all other plants under combined drought and salt stresses (Figure 2b). The photosynthetic rate of OA plants was 67%, 30%, and 20% higher than that of WT, A, and O plants, respectively, under this stressful condition. The combination of drought and salt led to a drop in photosynthetic rates for all plants when compared with those under the normal growth condition. The results showed that the photosynthetic rates of OA plants dropped by an average of 17% compared with that under normal growth condition; however, it dropped by 51% in wild‐type plants under combined drought and salt stresses (Figure 2b).

Analysis of relative water content (RWC) of these plants showed that OA plants maintained an average RWC of 70% compared to 49% in WT plants under combined drought and salt stresses. Moreover, the RWC of OA plants was 43%, 20% and 24% higher than that of WT, A and O plants, respectively (Fig. S1B). Higher photosynthetic rates in OA plants resulted in higher yield under combined drought and salt stresses. We did not observe significant differences among different genotypes under normal growth conditions; however, under combined drought and salt stresses, OA plants produced more bolls than all other genotypes (Fig. S1C). The total seed fibre yields produced by OA plants were 79%, 27% and 35% higher than those of WT, A and O plants, respectively (Figure 2c). OA plants also produced the largest root systems (Figure 2d), and their root biomass was 107% higher than that of WT plants under combined drought and salt stresses (Figure 2e).

### *OsSIZ1/AVP1* co‐overexpressing plants produced the highest yield under combined drought and heat stresses

**Figure 3:**
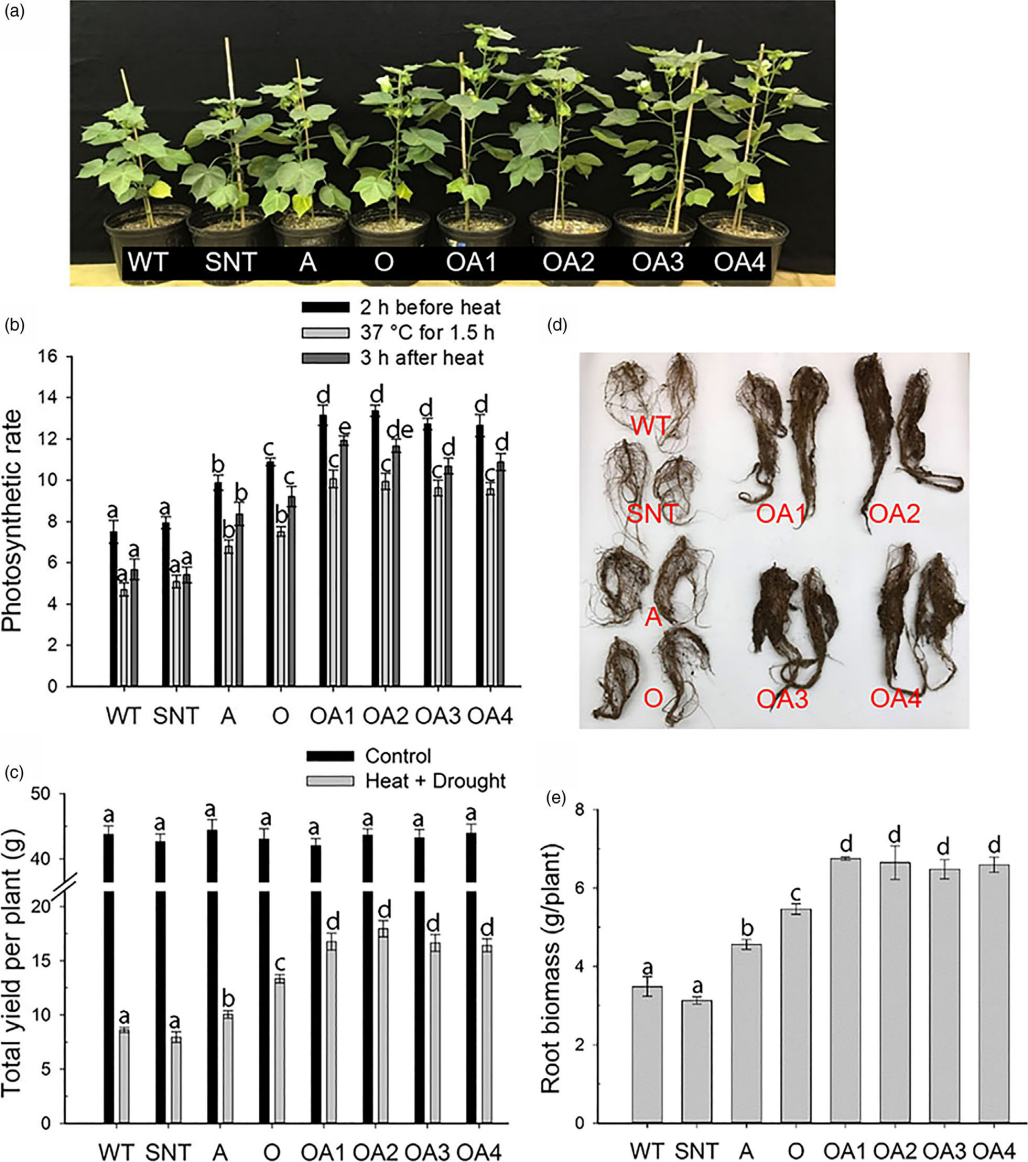
The effects of combined drought and heat stresses on control and *OsSIZ1/AVP1* co‐overexpressing cotton plants. (a) The phenotype of cotton plants six weeks after combined drought and heat treatment. (b) Photosynthetic rates of cotton plants two hours before heat treatment (black bar), during heat treatment at 37 °C (light grey bar), and 3 h after heat treatment (dark grey bar). (c) Total seed fibre yields under normal irrigation (black bar) and combined drought and heat stresses (grey bar). Results are the means ± SE (*n* = 6). (d) Root phenotypes of cotton plants under combined drought and heat stresses. (e) Root biomass analysis of cotton plants under combined drought and heat stresses. Results are the means ± SE (*n* = 6). WT, wild‐type plant; SNT, segregated non‐transgenic plant; A, *AVP1*‐overexpressing plant; O, *OsSIZ1*‐overexpressing plant; OA1 to OA4, four independent *OsSIZ1/AVP1* co‐overexpressing plants. Samples denoted by different letters are significantly different (*P* < 0.05, Tukey correction).

Combined drought and heat stresses are the most common combination of stresses in the arid and semiarid regions of the world. In this study, the performance of OA plants under combined drought and heat stresses was examined. Although no phenotypic differences were observed among different genotypes before the treatment of combined drought and heat stresses, OA plants displayed the best phenotype (i.e. being larger and taller) under combined drought and heat stresses (Figure 3a). Photosynthetic rates were measured six weeks after the initiation of combined drought and heat stresses in the growth chamber. Our measurements showed that OA plants displayed 72% higher photosynthetic rates than WT plants two hours before the start of heat stress and 108% higher photosynthetic rates during heat stress (Figure 3b). When the temperature increased to 37°C, the photosynthetic rates dropped significantly in all plants, but the drop was more dramatic in WT plants compared to OA plants. Our results showed that under heat stress, the photosynthetic rates were reduced by 37% and 24% in WT and OA plants, respectively. Although photosynthesis did not fully recover three hours after the heat stress ended, OA plants maintained 98% higher photosynthetic rates than WT plants during that period (Figure 3b).

Analysis of RWC in cotton plants showed that OA plants maintained an average RWC of 69% compared with 44% in WT plants under combined drought and heat stresses (Fig. S2A). The RWC in OA plants was 55%, 32% and 16% higher than that of WT, A and O plants, respectively. Under normal growth conditions, no differences in boll number and seed fibre yield were found among different genotypes. However, under combined drought and heat stresses, OA plants produced the largest number of bolls (Fig. S2B) and the highest seed fibre yields (Figure 3c) compared to all other plants. OA plants produced 97%, 53% and 37% more seed fibre than that of WT, A and O plants, respectively (Figure 3c). Combined drought and heat stresses resulted in severe reductions in seed fibre yields in all plants, but the drop was more significant in WT plants (Figure 3c). Root biomass analysis showed that OA plants produced the largest root systems (Figure 3d), with an average of 78% higher biomass in OA plants than WT plants under this combination of stresses (Figure 3e).

### Co‐overexpression of *OsSIZ1* and *AVP1* significantly increased the fibre yield of field‐grown cotton under the dryland conditions

Two field trials were conducted in 2016 and 2017 at the Experimental Farm of USDA‐ARS Cropping Systems Research Laboratory in Lubbock, Texas, to test the performance of OA plants under full‐irrigation and rain‐fed (i.e. dryland) conditions. Lubbock area receives an annual precipitation of 430 mm historically, and it is considered a semiarid region (Table S2). We evaluated the performance of OA plants under two different irrigation schemes: 15 mm of irrigation per week (full‐irrigation) and no‐irrigation (rain‐fed).

**Figure 4:**
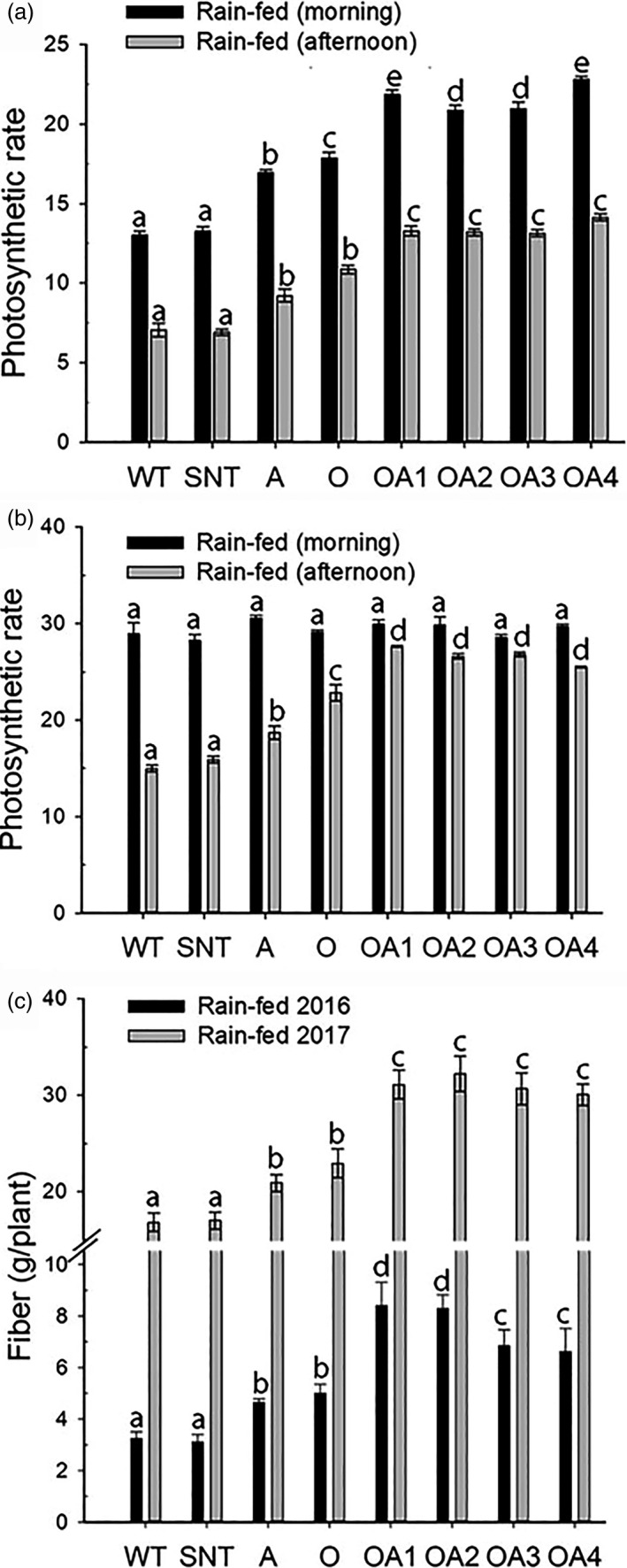
The performance of control and *OsSIZ1/AVP1* co‐overexpressing cotton plants under rain‐fed conditions in 2016 and 2017. (a) Photosynthetic rates of cotton plants under the rain‐fed condition in 2016. (b) Photosynthetic rates of cotton plants under the rain‐fed condition in 2017. Results are the means ± SE (*n* = 8). (c) Fibre yield produced per plant under the rain‐fed conditions in 2016 (black bar) and 2017 (grey bar). Results are the means ± SE. WT, wild‐type plant; SNT, segregated non‐transgenic plant; A, *AVP1*‐overexpressing plant; O, *OsSIZ1*‐overexpressing plant; OA1 to OA4, four independent *OsSIZ1/AVP1* co‐overexpressing plants. Samples denoted by different letters are significantly different (*P* < 0.05, Tukey correction).

The photosynthetic measurements of dryland grown cotton plants were taken in the morning and afternoon. The 2016 data showed that OA plants under rain‐fed condition displayed 66% and 88% higher photosynthetic rates than that of WT plants in the morning and in the afternoon, respectively (Figure 4a). In 2017, however, the morning photosynthetic rates did not show any differences among different genotypes under the rain‐fed condition, yet the afternoon measurements displayed 78% higher photosynthetic rates in OA plants than that in WT plants (Figure 4b). However, no differences in photosynthetic rates were found among different genotypes under the full‐irrigation conditions in 2016 and 2017 (Fig. S3A & B).

In 2016, OA plants produced 96% more bolls and 143% more total seed fibre per plant than WT plant under rain‐fed condition (Fig. S4A & B). In addition, the amount of fibre produced by OA plants was 133% higher than that by WT plant (Figure 4c), while A and O plants produced around 43% and 54% more fibre than that of WT plant (Figure 4c). In 2017, OA plants produced 83% more bolls and 84% total seed fibre per plant than those of wild‐type plants under rain‐fed condition (Fig. S4C & D). The amount of fibre produced by OA plants was 81% higher than that of WT plant, while A and O plants produced 24% and 36% more fibre than WT plant did (Figure 4c).

Fibre quality of cotton plants grown in the field under rain‐fed condition was analysed using the High Volume Instrument (HVI) method. Because one of the most important characteristics of fibre quality is the maturity of fibre, which affects the length, strength and fineness of cotton fibre (Ayele *et al*., 2017); therefore, we analysed micronaire, length and strength of the cotton fibre in WT, A, O and all four OA plants. Our results exhibit some variations in micronaire, length and strength of fibre across all genotypes (Fig. S5).

### *OsSIZ1/AVP1* co‐overexpressing cotton plants use water most efficiently

**Figure 5:**
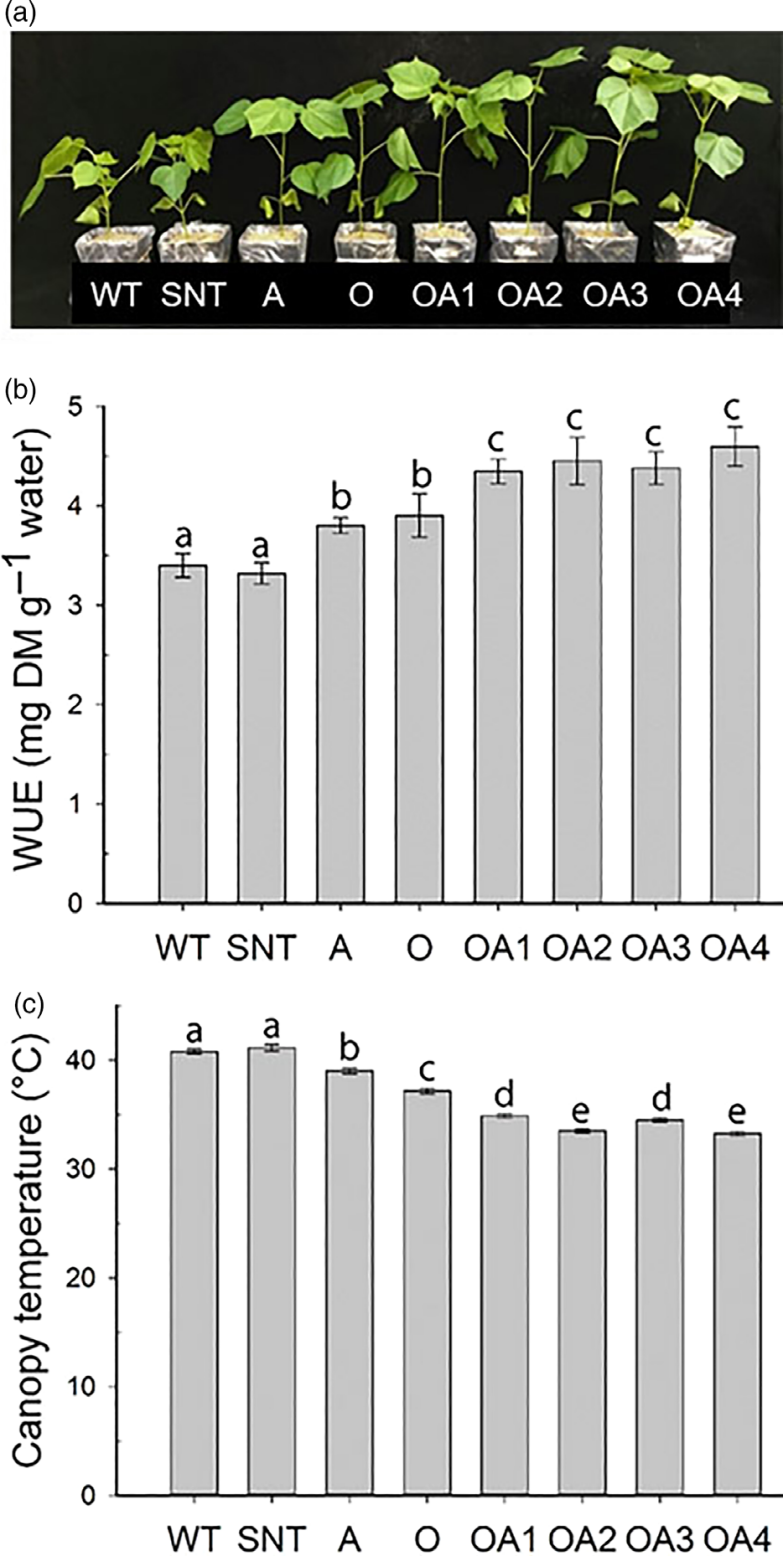
Analysis of water use efficiency and canopy temperature of control and *OsSIZ1/AVP1* co‐overexpressing cotton plants. (a) Phenotypes of cotton plants twenty‐five days after irrigating plants with an initial equal volume of water. (b) Water use efficiency of cotton plants in biomass produced per gram of water consumed by plant. Data are the means ± SE (*n* = 10). (c) Canopy temperatures of control and *OsSIZ1/AVP1* co‐overexpressing cotton plants grown in field under the rain‐fed condition in 2017. Data are the means ± SE (*n* = 65). WUE, water use efficiency; WT, wild‐type plant; SNT, segregated non‐transgenic plant; A, *AVP1*‐overexpressing plant; O, *OsSIZ1*‐overexpressing plant; OA1 to OA4, four independent *OsSIZ1/AVP1* co‐overexpressing plants. Samples denoted by different letters are significantly different (*P* < 0.05, Tukey correction).

To analyse how efficiently OA plants would use water during seedling growth, we measured the water use efficiency (WUE) of these plants using the protocol of Xin *et al*. (2008). The results showed that OA plants produced the biggest biomass than all other plants with equal amount of water provided (Figure 5a). OA plants displayed 30% higher WUE than WT plant (Figure 5b), indicating that co‐overexpression of *OsSIZ1* and *AVP1* helps plants use water more efficiently than WT, SNT, A and O plants.

The canopy temperature is an important indicator of heat stress for plants under water‐deficit conditions in the summer. If plants can absorb water more efficiently, then they would be able to cool down leaf temperatures more effectively, thereby maintaining healthier cellular metabolism. We measured the canopy temperature of rain‐fed grown cotton plants in the field. Our data indicated that OA plants had a 16% lower leaf temperature compared to WT plants (Figure 5c). This result is consistent with the water use efficiency of OA plants, which explains why OA plants performed the best under water‐deficit conditions in the summer.

### Co‐overexpression of *OsSIZ1* and *AVP1* improves plant performance under low and high phosphorus conditions

**Figure 6:**
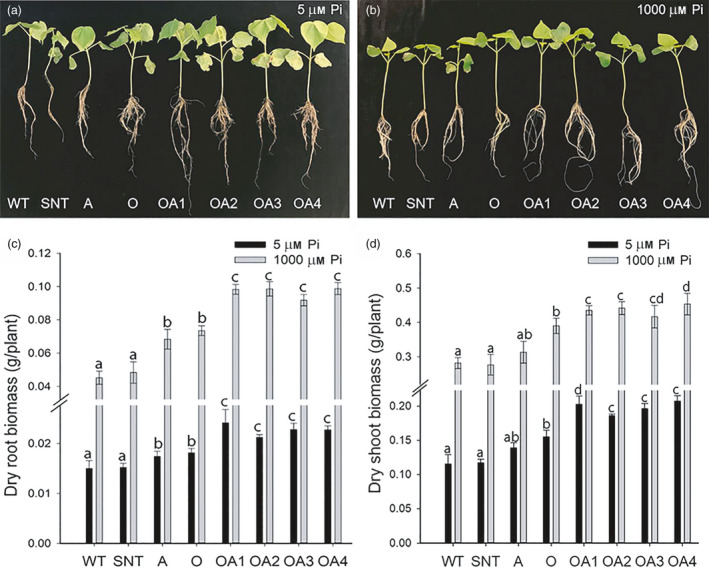
Phenotype and biomass analysis of control and *OsSIZ1/AVP1* co‐overexpressing cotton plants grown in hydroponic condition under different concentrations of inorganic phosphorus. (a) Phenotype of control and *OsSIZ1/AVP1* co‐overexpressing cotton plants under low phosphate concentration (5 µm KH_2_PO_4_). (b) Phenotype of control and *OsSIZ1/AVP1* co‐overexpressing cotton plants under high phosphate concentration (1000 µm KH_2_PO_4_). (c) Dry‐root biomass of control and *OsSIZ1/AVP1* co‐overexpressing cotton plants grown under low (black bar) and high phosphate (grey bar) concentrations. (d) Dry‐shoot biomass of control and *OsSIZ1/AVP1* co‐overexpressing cotton plants grown under low (black bar) and high phosphate (grey bar) conditions. Results are the means ± SE (*n* = 6). WT, wild‐type plant; SNT, segregated non‐transgenic plant; A, *AVP1*‐overexpressing plant; O, *OsSIZ1*‐overexpressing plant; OA1 to OA4, four independent *OsSIZ1/AVP1* co‐overexpressing plants. Samples denoted by different letters are significantly different (*P* < 0.05, Tukey correction).

A previous study by Sawan *et al*. (2008) showed that phosphorus promotes uptake of potassium and nitrogen, increases chlorophyll content and dry biomass in cotton. The phenotypes of cotton plants grown under low and high phosphorus conditions (i.e. 5 µm KH_2_PO_4_ and 1000 μm KH_2_PO_4_, respectively) clearly showed that OA plants displayed the best phenotype among all genotypes (Figure 6a & b). Under high phosphorus concentration, the dry‐root biomass of OA plants was 114% higher than that of WT plant, while A and O plants were 42% and 32% higher than that of WT plant (Figure 6c). Under low phosphorus concentration, the dry‐root biomass of all plants dropped significantly, yet the dry‐root biomass of OA plants was 52%, 31% and 26% higher than that of WT, A and O plants, respectively (Figure 6c). Under high phosphorus concentration, the dry‐shoot biomass of OA plants was 55% higher than that of WT plant, whereas O plants and A plants were 40% and 12% higher than that of WT plants (Figure 6d). Under the low phosphorus concentration, OA plants again demonstrated the highest dry‐shoot biomass compared to all other plants. The results showed that the dry‐shoot biomass of OA plants was 72%, 42% and 28% higher than that of WT, A and O plants, respectively (Figure 6d). These results indicate that co‐overexpression of *OsSIZ1* and *AVP1* in cotton promotes plant growth and development under low and high concentrations of phosphorus.

### Identification of differentially expressed genes in *OsSIZ1/AVP1* co‐overexpressing plants grown in the field

**Figure 7:**
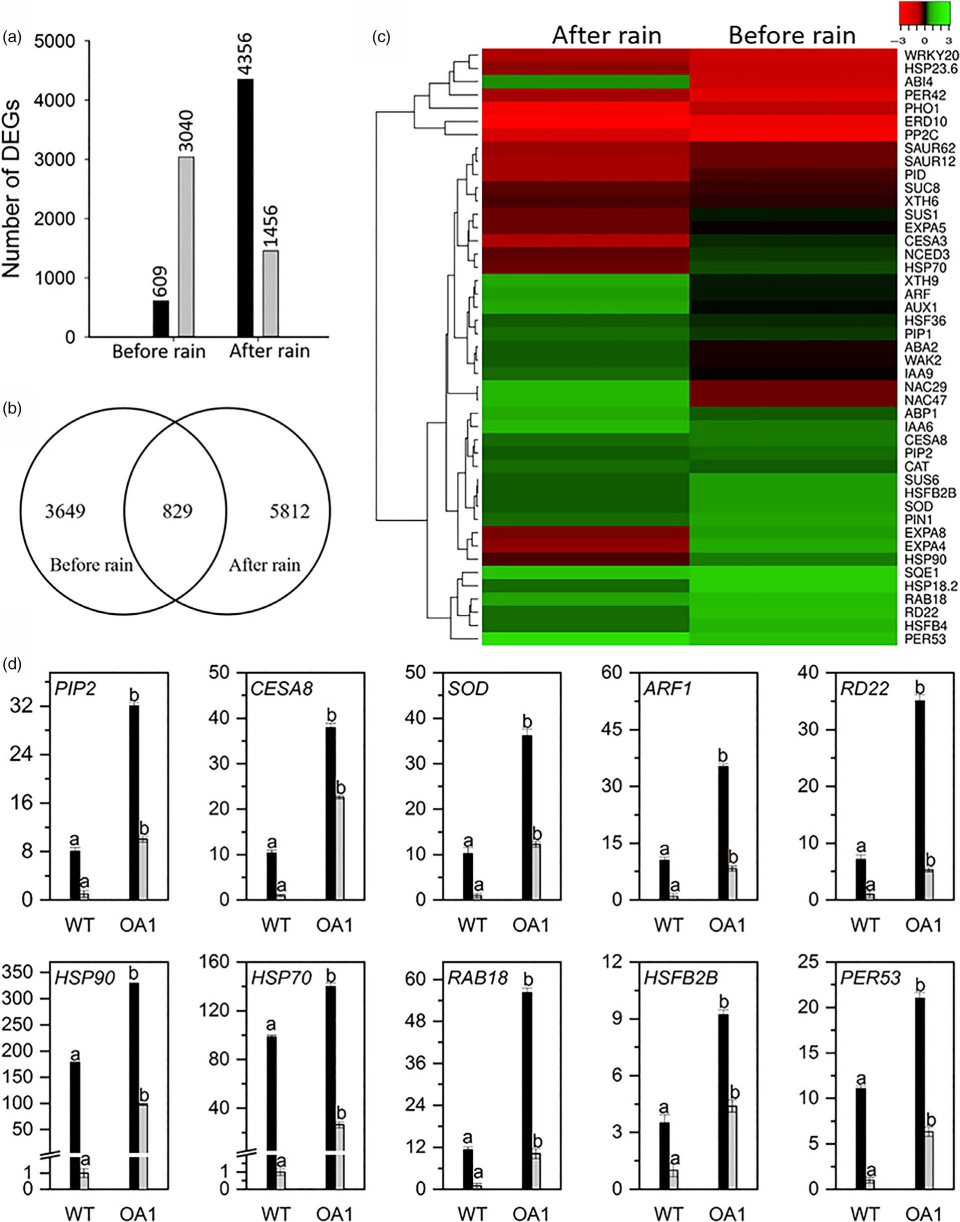
RNA‐sequencing analysis of control and *OsSIZ1/AVP1* co‐overexpressing cotton plants in the field before and after rain. (a) Numbers of differentially expressed genes before and after rain. Black bars represent down‐regulated genes, and grey bars represent up‐regulated genes in *OsSIZ1/AVP1* co‐overexpressing plants vs. wild‐type plants before and after rainfall. (b) Venn diagram of transcripts showing differential expression in leaf tissues of *OsSIZ1/AVP1* co‐overexpressing plants vs. wild‐type plants before and after rain. (c) Heatmap of 45 differentially expressed genes in *OsSIZ1/AVP1* co‐overexpressing plants compared to wild‐type plants before and after rain. (d) Quantitative real‐time PCR analysis of ten stress‐related genes in *OsSIZ1/AVP1* co‐overexpressing plants vs. wild‐type plants before rain (black bar) and after rain (grey bar). Data are means ± SE (*n* = 3). DEGs, differentially expressed genes; WT, wild‐type plant; OA1, *OsSIZ1*/*AVP1* co‐overexpressing plant 1. Samples denoted by different letters are significantly different (*P* < 0.05, Tukey correction).

A comparative transcriptomic analysis was conducted on rain‐fed grown OA1 and WT plants before and after rainfall to investigate the underlying mechanism of the superior performance of OA plants in the field. The RNA‐sequencing data showed that there were 3649 differentially expressed genes (DEGs) in OA1 compared to WT before rainfall, among which 609 were down‐regulated, and 3040 were up‐regulated. However, after precipitation, a total of 5812 DEGs were found in OA1 with 4356 down‐regulated and 1456 up‐regulated genes (Figure 7a).

The Venn diagram provides an overview of the distribution of DEGs in OA1 plants compared to WT plants before and after precipitation. OA1 plants share 829 DEGs that were either up‐regulated or down‐regulated before and after rainfall (Figure 7b). Among those shared 829 DEGs, we selected 45 DEGs with the fold‐of‐change > 2 for further analysis. Transcriptomic analysis showed that stress‐induced heat‐shock protein (HSP) genes (e.g. *HSP70* and *HSP90*) were up‐regulated in field‐grown OA1 plants compared to WT before rainfall, with higher levels of transcripts (log_2_‐fold > 1.1; Figure 7c). Also, transcripts from two members of the small heat‐shock protein genes, *sHSP20* and *sHSP18*.6, were highly up‐regulated in the OA1 plant with log_2_‐folds of 7.8 and 15.6, respectively. However, the transcripts of these HSP genes were down‐regulated during the recovery stage after rainfall, indicating important roles of heat‐shock proteins as molecular chaperons under field drought conditions (Wang *et al*., 2004). Transcriptional factors (TFs) are critically important in plant response to environmental stresses such as heat and drought. Our data showed that the transcript levels of two heat‐shock TF genes *HSFB4* and *HSFB2B* were up‐regulated in OA1 before rain by 2.5 and 107.15 folds, respectively. The transcript levels of some ABA‐dependent and drought stress‐related genes such as *RD22*, *NCED3* and *RAB18* were also highly up‐regulated in OA1 plants before the rain.

Several cell wall‐associated genes were up‐regulated in OA1 plants before the rain, such as cellulose synthase genes *CESA3* and *CESA8*. These genes are required in the biosynthesis of primary and secondary cell walls, therefore needed for building thicker cell wall that could withstand negative pressure under drought stress condition (Chen *et al*., 2005). The transcript of the gene *xyloglucan endotransglucosylase/hydrolase 6* (*XTH6*) was shown to be up‐regulated in OA1 plants, indicating a potential role of this hydrolase in cell wall remodelling in response to water deprivation (Tenhaken, 2015). Transcripts of two expansin genes *EXPA4* and *EXPA8* were also up‐regulated under drought stress conditions, which is consistent with previous reports (Han *et al*., 2012; Tenhaken, 2015). The *Squalene epoxidase 1* (*SQE1)* is another gene whose transcript level was significantly up‐regulated (log_2_‐fold > 15.4).

Auxin‐mediated signalling pathway promotes transcription of genes that encode plasma membrane ATPase, K^+^ channels, expansins and cell wall remodelling enzymes (Wang and Ruan, 2013). The transcriptome analysis revealed that transcripts of several auxin‐related genes such as *pinformed 1* (*PIN1*), *auxin transporter‐like 1* (*AUX1*), *auxin binding protein 1* (*ABP1*) and *auxin‐responsive factor* (*ARF*) are highly up‐regulated in OA1 plants by log_2_‐fold changes of 8.2, 5.4, 7.2 and 3.2, respectively. Sucrose, a primary sugar source in higher plants, is converted to glucose and fructose by sucrose synthase (SUS). The activities of the SUS family enzymes are crucial for cellulose synthesis in plants. SUS was proposed to be involved in cell wall thickening and cotton fibre development (Zou *et al*., 2013), cell expansion (Wang and Ruan, 2013), sugar import (Klotz *et al*., 2003) and environmental stress response (Bieniawska *et al*., 2007). Besides, lower SUS activity in transgenic cotton plants negatively affects fibre initiation and development (Ruan *et al*., 2003). Our results showed that transcript of *SUS6* was up‐regulated (log_2_‐fold > 3.5) in OA1 plants before the rain. The transcriptome analysis data presented here are in agreement with recently proposed regulatory roles for auxin and sugar signalling in cell division and proliferation in both vegetative and productive stages of plant growth and development under drought stress conditions.

### Quantitative RT‐PCR analysis confirms RNA‐sequencing results

The validation of RNA‐sequencing data was conducted by quantitative real‐time PCR (qRT‐PCR) analysis. Ten stress‐related genes were selected for qRT‐PCR analysis using gene‐specific primers (Table S3), and the cotton *UBQ7* was used as the internal reference gene. The results from qRT‐PCR experiments showed a similar transcript pattern with the RNA‐sequencing data (Figure 7d): the transcript levels of these genes were highly up‐regulated before the rain (i.e. under drought stress condition) when compared to those after rain. Our results indicate that co‐overexpression of *OsSIZ1* and *AVP1* in cotton might activate different stress signalling pathways, leading to up‐regulation of transcript levels of stress‐related genes such as *RD22, HSFB2B, HSP70, HSP90*, *NCED3*, *RAB18, SOD, CESA8*, *XTH6,* resulting in significantly increased tolerance to environmental stresses in transgenic plants.

## Discussion

Crops usually grow in suboptimal conditions, which prevents them from achieving their full growth and reproduction potential. According to Atkinson and Urwin (2012), plant response to multiple stresses is different from response to individual stresses, and the responses are not simply additive or subtractive. Based on climate prediction models, the surface temperature will likely increase by 3–5 °C within the next 50–100 years, which will lead to more unpredictable weather patterns such as more frequent drought or flood in many regions of the world, thereby adversely affecting agricultural productivity (Solomon *et al*., 2007). The concurrence of changing climate with the rapid growth of the world population poses a serious threat to world food security. Hence, the need for stress‐tolerant crops that can meet the future global demands for food and fibre has not been greater than today. It is estimated that world food production must increase by 70%–100% to meet the need of the growing population (Edgerton, 2009). Conventional breeding could be, in fact, has been used to improve crop tolerance to abiotic stresses, but it might take longer time to develop stress‐tolerant crops via this approach. Furthermore, developing multi‐stress‐tolerant crops through a traditional approach has not been very successful; thus, we need new approaches to address this problem. About two decades ago, scientists started using the recombinant DNA technology to improve crop production, and many genes that confer increased tolerance to various abiotic stresses were discovered and tested. In this study, we generated a transgenic cotton population that co‐overexpress *OsSIZ1* and *AVP1*, and we show that this approach appears to be very effective in making transgenic cotton significantly more stress‐tolerant.

Overexpression of *AVP1* was shown to improve drought and salt tolerance in transgenic plants such as Arabidopsis, tomato, cotton and peanut (Gaxiola *et al*., 2001; Gaxiola *et al*., 2012; Pasapula *et al*., 2011; Qin *et al*., 2013). Overexpression of *OsSIZ1* was shown to improve drought and heat tolerance in transgenic creeping bentgrass, cotton and Arabidopsis (Li *et al*., 2013; Mishra *et al*., 2018; Mishra *et al*., 2017). We hypothesized that co‐overexpression of *OsSIZ1* and *AVP1* might further improve tolerance to multiple stresses, leading to substantially higher yield. Recently, we tested this hypothesis by co‐overexpressing these two genes in Arabidopsis, and we showed that indeed co‐overexpression of *AVP1* and *OsSIZ1* in Arabidopsis could improve plant tolerance to multiple stresses and significantly increase seed yields under single stress and multiple stress conditions (Esmaeili *et al*., 2019).

In this study, we further extended our finding to a real crop, the upland cotton. Our results firmly prove that co‐overexpression of *OsSIZ1* and *AVP1* significantly improves cotton's tolerance to combined drought/salt and combined drought/heat stresses with a significant increase in fibre yields (Figures 2 and 3). In addition, our results from field‐grown plants were consistent with the results from plants grown in greenhouse and growth chambers in that OA plants performed the best under stress conditions. In the field study of 2016, OA plants produced 133% more fibre than WT plants (Figure 4c). In 2017, there was a big variation in the total seed fibre yield as well as in the amount of fibre produced per plant, which was partly due to the higher rainfall that Lubbock area received during the cotton growing season (Table S2). Despite this, OA plants still produced 81% more fibre than WT plants under the rain‐fed condition (Figure 4c). Based on the two years' field studies, the average fibre yield of OA cotton plants is 107% higher than that of wild‐type cotton plants.

The dramatic improvement of abiotic stress tolerance in OA plants is likely due to a synergistic interaction between overexpression of *AVP1* and overexpression of *OsSIZ1*, which leads to a better physiological state compared to overexpression of *AVP1* or *OsSIZ1*, separately. To better understand the molecular mechanism underlying the significant improvement in stress tolerance and yield increase in OA plants, we conducted a transcriptome analysis on field‐grown OA and WT plants before rain (under drought stress) and during the recovery stage after rain. Our RNA‐sequencing results showed a complex mechanism in OA plants in response to field drought conditions, which might involve many vital players that include transcriptional factors, hormones, heat‐shock proteins, cell wall biosynthesis and remodelling enzymes, as well as antioxidant enzymes.

As molecular chaperones, HSPs stabilize proteins under various stress conditions. HSPs were shown to be involved in response to combined heat and drought stresses in maize and wheat (Grigorova *et al*., 2011; Hu *et al*., 2010). Heat‐shock factors (HSFs) are transcriptional factors that control the expression of HSP genes. Here, we found that the transcript level of *HSFB2B* is significantly up‐regulated in OA plants before the rain (Figure 7d), indicating that this gene may play an important role in OA plants in response to the field stress of drought plus summer heat (Ikeda *et al*., 2011). Moreover, transcript levels of another heat‐shock TF gene *HSFB4* and several HSP genes such as *HSP70* and *HSP90* were also up‐regulated in OA plants before the rain (Figure 7d).

Reactive oxygen species (ROS) are by‐products of cellular metabolism in plants, which are usually over‐produced under abiotic stresses, creating a detrimental condition called oxidative stress. Plants, however, developed effective mechanisms (e.g. antioxidant molecules and antioxidant enzymes) to respond to oxidative stress and minimize the damages caused by ROS (Atkinson and Urwin, 2012). Previously, Mishra *et al*. (2017) showed that transcript levels of antioxidant genes such as *APX*, *SOD* and *GS* were increased in cotton plants under combined heat and drought stresses. In this study, OA plants showed higher transcript levels for superoxide dismutase gene (*SOD*) and peroxidase 53 gene (*PER53*) under field drought condition (Figure 7d). Therefore, maintaining homeostasis of ROS by regulating the expression of antioxidant genes increases plant tolerance to a variety of environmental stresses.

Drought tolerance in plants can be achieved by lowering the transpiration rate via closing stomata and by increasing ABA sensitivity (Aroca *et al*., 2006). The responsive to dehydrin 22 gene (*RD22*) is a drought inducible and ABA inducible gene (Abe *et al*., 2003), its transcript was highly up‐regulated in OA plants under field drought condition (Figure 7d). *RAB18* is another ABA‐regulated gene (Valliyodan and Nguyen, 2006), and its transcript level was also significantly up‐regulated in OA plants (Figure 7d). Furthermore, we found that transcript levels of several protein phosphatase 2C (PP2C) genes that negatively regulate ABA signalling were down‐regulated in OA plants (Figure 7c). This result is in agreement with previously reported data in cotton under drought stress conditions (Hou *et al*., 2018). Transcript levels of other ABA‐regulated genes like *NCED3* (Satoshi *et al*., 2001) were also significantly increased in OA plants (Figure 7c), indicating that the ABA‐dependent pathway is involved in the improved drought tolerance in OA plants.

Phosphorous is an essential element in plants that plays pivotal roles in serving as a component of DNA and protein, and in energy metabolism. Previous reports showed that *Phosphate Transporter 1, PHO1,* is expressed in vascular tissues of Arabidopsis roots, and it functions in phosphorous transfer into the apoplastic space of xylem vessels (Hamburger *et al*., 2002). *PHO1* is also expressed in guard cells, and it contributes to ABA‐induced stomatal closure and reduces transpiration under drought stress conditions. It was also demonstrated that ABA‐induced gene expression is not affected by the down‐regulation of *PHO1* (Zimmerli *et al*., 2012). In this study, we found that the transcript level of *PHO1* was less down‐regulated in OA plants under drought stress (Figure 7c).

The proteins PIP1 and PIP2 belong to the subfamily of plasma membrane intrinsic proteins (PIPs) with water channel activity, and we show here that their transcript levels were increased in OA plants under field drought conditions (Figure 7d). This result is in agreement with a previous study by Park *et al*. (2012), indicating the involvement of aquaporins in water‐deficit stress response in cotton. Recently, a genome‐wide association study on cotton revealed that *PIP2* plays a distinctive role in drought tolerance (Hou *et al*., 2018). In addition to their function in intercellular water transport, aquaporins are also involved in leaf CO_2_ conductivity (Heckwolf *et al*., 2011; Uehlein *et al*., 2003). Although the roles of PIPs in drought stress tolerance are not yet fully understood, improved drought tolerance was achieved in tobacco and rice plants that overexpress *PIP1* (Ferrante *et al*., 2006).

Squalene epoxidase is an enzyme that converts squalene to 2,3‐oxidosqualene, a precursor in the sterol biosynthetic pathway in plants. It was shown that a mutation in *SQE1* reduces root and hypocotyl elongation (Posé *et al*., 2009). In addition, the *seq1‐5* mutant showed an extreme drought sensitivity due to its inability to modulate stomatal closure (Posé *et al*., 2009). Here, we demonstrated that the transcript level of *SQE1* was significantly up‐regulated (log_2_‐fold > 15.4) in OA plants under field drought condition (Figure 7c), indicating a positive role of this gene in drought tolerance of OA plants.

Cell wall plays a crucial role in providing mechanical strength and cell shape in plants, and it has been well established that cell wall remodelling occurs under abiotic stress conditions (Tenhaken, 2015). Expansins (EXP) and xyloglucan endotransglucosylases/hydrolases (XTH) modulate the interactions among cell wall components (Tenhaken, 2015). The transcriptome analysis showed that the transcript levels of several cell wall‐related genes (i.e. *XTH6*, *EXPA4*, and *EXPA8*) were up‐regulated in OA plants under field drought conditions (Figure 7c). The involvement of these genes in drought tolerance in plants was reported by Lee *et al*. (2001) and Rose *et al*. (2002).

Our transcriptome analysis showed the up‐regulation of transcripts from several auxin‐related genes such as *PIN1*, *AUX, ARF* and *ABP1* in OA plants as well (Figure 7c & d). Auxin binding protein 1 (ABP1) is an auxin receptor that perceives extracellular auxin. ABP1 activates the plasma membrane H^+^‐ATPase when it interacts with several membrane‐associated proteins. This interaction results in lower cell wall matrix pH, which in turn relaxes cell wall for expansion via activation of cell wall loosening proteins such as expansins and XTH (Wang and Ruan, 2013). A previous study in tobacco showed that reduced expression of *ABP1* inhibits auxin‐induced cell elongation (Chen *et al*., 2001). However, the activation of plasma membrane H^+^‐ATPase results in osmotically driven water uptake for expansion by activating the voltage‐dependent potassium inward channels (Wang and Ruan, 2013). Overexpression of *AVP1* in Arabidopsis increases auxin polar transport and contributes to lowering apoplastic pH by modulating PIN1 (Li *et al*., 2005). Auxin also contributes to fibre development and boll retention in cotton via abscission regulation (Lee *et al*., 2007). Up‐regulation of several auxin‐related genes in OA cotton plants contributes to the improved performance and increased boll number and fibre yield in OA plants under rain‐fed condition. In addition, the accumulation of osmolytes, ions and sugars in plant cells maintains a lower water potential to increase water flux into plant cells. This generates higher turgor pressure within plant cells and leads to cell expansion. The up‐regulated transcripts of several SUS family genes such as *SUS6* might also contribute to the enhanced phenotype of OA plants by maintaining higher net internal turgor pressure. Thus, the interplay of auxin and sugar signalling pathways plays an important role in plant growth and development (Wang and Ruan, 2013).

We also found that the transcripts of *CESA3* and *CESA8* were up‐regulated in OA plants under field drought conditions. These two genes are members of the cellulose synthase gene family that is involved in primary and secondary cell wall biosynthesis, and mutations in these genes affect the formation of xylem that is responsible for water transport from root to shoot. One study showed that cellulose synthase genes were down‐regulated in cotton roots under drought stress condition and thereby, plants could allocate sugars towards osmoprotectants instead of cell wall biosynthesis, which in turn results in xylem collapse and impedes water transport in plant (Singh *et al*., 2016). However, a comparative transcriptomic analysis of roots of *Gossypium herbaceum* showed that the cellulose synthase genes were highly expressed in tolerant genotypes under drought stress condition (Ranjan *et al*., 2012). Another study by Rasheed *et al*. (2016) indicated that the transcript levels of some cellulose synthase genes including *CESA8* were up‐regulated in Arabidopsis under drought stress condition, suggesting that increased expression of some members of the cellulose synthase gene family like *CESA8* might contribute to the improved drought tolerance, which is consistent with what we found in this study.

**Figure 8:**
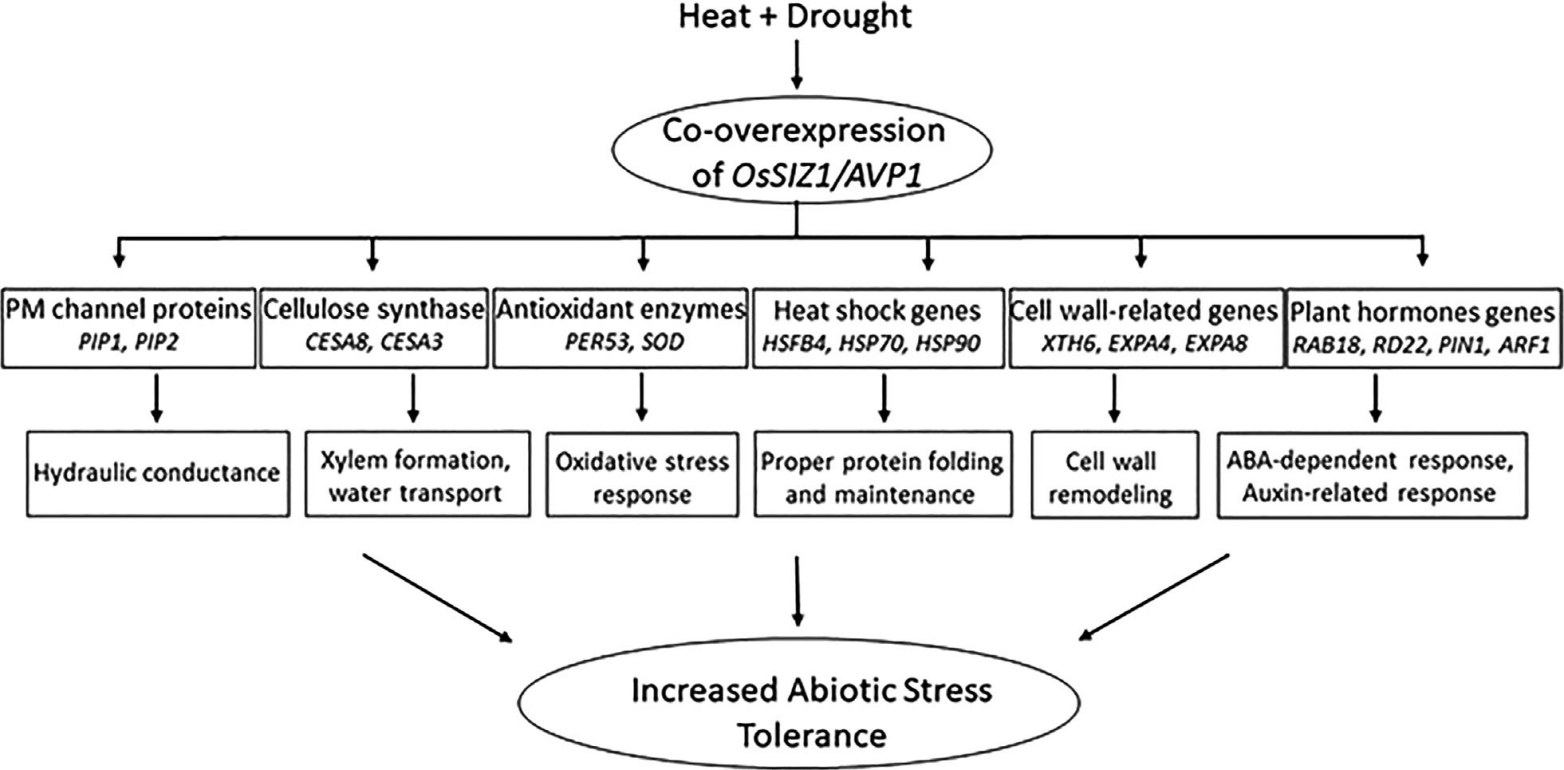
Proposed model to illustrate the potential molecular mechanisms behind the increased abiotic stress tolerance in *OsSIZ1/AVP1* co‐overexpressing cotton plants under rain‐fed conditions. Under drought and heat stress conditions, co‐overexpression of *OsSIZ1* and *AVP1* in cotton leads to up‐regulation of genes involved in cell wall biosynthesis, cell wall remodelling, antioxidation metabolism, plant hormone signalling pathways, and protein homeostasis, which in turn results in increased tolerance to abiotic stresses and higher yields under dryland conditions.

Our transcriptomic data indicate that co‐overexpression of *OsSIZ1* and *AVP1* triggers sophisticated changes in the transcriptional regulatory networks, which enables transgenic plants to withstand complex environmental stress conditions. Although our transcriptomic analysis may not cover all possible mechanisms for the increased stress tolerance in the OA plants, the Figure 8 is a summary of the molecular mechanisms that we identified responsible for the dramatic improvement in abiotic stress tolerance in OA plants. This research is the first example in a real crop plant, showing that we can make transgenic cotton to tolerate multiple abiotic stresses simultaneously, and the tolerance level to multiple stresses is much higher than any previously reported studies. For example, we had seen an increase of 20% to 25% in photosynthetic rates or yields in some of the most successful studies in literature, we rarely saw studies with higher than 30% increase in photosynthetic rates or yields in transgenic plants vs. wild‐type plants, while an increase of 80% or over 100% in yield has not been reported previously. Our data from experiments conducted in greenhouse, growth chamber and field all support the conclusion that *OsSIZ1/AVP1* co‐overexpression will likely lead to doubling of yield for crops grown in dryland conditions as well as in semiarid and arid regions of the world.

## Conclusion

Currently, the genetic engineering approach seems incapable of overcoming the negative impact of multiple abiotic stresses on plants, if just based on manipulation of single gene overexpression in transgenic plants. It is evident that more than one gene is required to generate a successful stress‐tolerant crop that can withstand a variety of environmental stresses. This research provides a paradigm for a genetically modified crop that can grow successfully in the field under multiple stress conditions. Our results demonstrate that co‐overexpression of *OsSIZ1* and *AVP1* not only improves transgenic cotton yield compared to wild‐type cotton under multiple stress conditions but also, it does not negatively affect plant yield when there is no stress. We believe that this would be a revolutionary approach that helps secure future food and fibre need for mankind.

## Material and methods

### Vector construction and cotton transformation

The p35S::AVP1/pUbi::OsSIZ1 construct harbouring *NptII* (kanamycin resistance gene) was cloned into the pBI121‐based binary vector as described by Esmaeili *et al*. (2019). The plasmid was then transformed into Agrobacterium strain GV3101 and the transformed Agrobacterium was used for cotton transformation. The upland cotton *Gossypium hirsutum* L. var Coker 312 was used for transformation using the protocol by Bayley *et al*. (1992).

### Plant materials

Wild‐type Coker 312, segregated non‐transgenic line, two reference lines *OsSIZ1*‐overexpressing line (Mishra *et al*., 2017) and *AVP1*‐overexpressing line (Pasapula *et al*., 2011), and four independent *OsSIZ1/AVP1* co‐overexpressing plants were used in this study. Homozygous T_4_ to T_6_ plants of each independent OA plants were used for experiments described in this study.

### RNA and DNA blot analyses

About 100 mg of thoroughly ground cotton leaves were used for total RNA extraction using the Spectrum^™^ Plant Total RNA Kit (Sigma). Twenty µg of total RNAs from each sample was used in the electrophoresis and blotted onto a nylon membrane. The membrane was hybridized with the P^32^‐labelled gene‐specific probes for *OsSIZ1*, *AVP1* and *Ubi7*, respectively, under the condition as described by Church and Gilbert (1984). The specific primers that were used to amplify the full cDNA of *OsSIZ1*, *AVP1* and *Ubi7* genes are listed in Table S1.

Genomic DNA isolation was carried out using the CTAB method with slight modifications (Paterson *et al*., 1993). Overnight digestion of 20 µg of genomic DNA from WT, SNT and four independent OA plants was carried out with the restriction enzyme *Hind* III, then separated by electrophoresis and blotted onto a nylon membrane. The DNA hybridization was conducted as previously described by Hu *et al*. (2014) using P^32^‐labelled gene‐specific probe for *NptII* (Table S1).

### Combined drought and salt treatment in greenhouse

Combined drought and salt stresses were started four weeks after seed germination by irrigating plants with 250 mL of saline water containing 50 mm NaCl every other day for six days, then the salinity was increased to 100 mm for another six days. Thereafter, plants were irrigated with 500 mL of plain water per pot every other day. Photosynthetic rates were measured one month after combined drought and salt stresses were started.

### Combined drought and heat treatment in growth chamber

Reduced irrigation was started four weeks after seed germination with 500 mL of water per pot every other day while the temperature of the growth chamber was set at 25 °C at night and 28 °C during the day, except from 1:00 pm to 3:00 pm, the temperature was increased to 37 °C. The chamber photoperiod was 16 h light/8 h dark, and the relative humidity was maintained around 60%. Three different photosynthetic measurements were taken each day: 2 h before the heat stress was started, during heat stress and 3 h after the heat stress.

### Relative water content analysis

Three leaf discs of the fourth leaf from the top were prepared and the fresh weight (FW) of discs was measured immediately. Then the leaf discs were immersed in distilled water overnight and turgid weight (TW) was measured the next day, then discs were dried in an oven set at 65 °C for two days. Then, the dry weights (DW) of samples were measured, and RWC was calculated using the formula below:

$$\text{RWC(\textbackslash{}\% )}=\left(\text{FW}\;‐\;\text{DW}\right)/\left(\text{TW}\;‐\;\text{DW}\right)\times 100.$$

### Field trials and fibre analysis

Two field trials were conducted in 2016 and 2017 at the Research Farm of USDA‐ARS Cropping Systems Laboratory in Lubbock, Texas. A total of 405 seeds per genotype were planted in 9 randomized blocks with 45 seeds in each individual blocks of the rain‐fed field. The field was surrounded by three rows of purple cotton to prevent leaking of moisture from neighbouring fields. The fully irrigated field was planted with the total of 180 seeds per genotype, which were randomized in four blocks of 45 seeds in each individual blocks. Photosynthetic rates were measured in the morning (9:00–11:00 am) and in the afternoon (2:00–5:00 pm) on the same day. The weather information for Lubbock area in 2016 and 2017 is provided in Table S2. At the end of the growth season, nine one‐metre plots of each genotype were analysed in both fully irrigated and rain‐fed conditions. The total number of bolls per plant, total seed fibre yield per plant and total lint produced per plant were analysed within each one‐metre plot. Cotton fibres from the field‐grown cotton plants were hand‐harvested and hand‐delinted at the end of 2017 growth season and then analysed at Fiber and Biopolymer Research Institute of Texas Tech University.

### Water use efficiency and canopy temperature measurement

The water use efficiency was determined as described by Mishra *et al*. (2017) in greenhouse using 10 days old plants, and WUE was calculated ten days after the initiation of the experiment. Canopy temperatures of rain‐fed grown plants were measured using an infrared thermometer as described by Mahan and Yeater (2008). The fourth top leaves of 65 plants per genotype were randomly selected for this experiment.

### The effect of low and high phosphorous on plant growth

In this hydroponic experiment, the basal salts solution was made of 2 mm Ca(NO_3_)_2_.4H_2_O, 1.25 mm NH_4_NO_3_, 0.1 mm KCl, 0.65 mm K_2_SO_4_, 0.65 mm MgSO_4_, 1 µm MnSO_4_, 10 µm H_3_BO_3_, 0.5 µm (NH_4_)_6_Mo_7_O_24_, 0.1 µm CuSO_4_.5H_2_O, 1 µMZnSO_4_.7H_2_O and 0.1 mm Fe‐EDTA. Two hydroponic solutions were prepared as basal salts with low concentration of phosphate (5 µm KH_2_PO_4_) and basal salts with high concentration of phosphate (1000 µm KH_2_PO_4_). The pH value of each solution was adjusted to 6.5 (Pei *et al*., 2012). Cotton seeds were surface sterilized and germinated on Stewart media for 4 days. Then seedlings were transferred to hydroponic solutions, and they were incubated on a rotary shaker in a growth chamber for three weeks. The dry biomass of root and shoot was analysed after treatment at 65 °C in an oven for 48 h.

### RNA‐sequencing and transcriptome analysis

A comparative transcriptomic analysis was performed on WT and OA1 plants grown in the field in the third week of July under drought stress condition (two weeks without precipitation with daytime temperatures over 35 °C) and 4 h after rain (recovery stage). Total RNAs were extracted from leaves using the Spectrum^™^ Plant Total RNA kit (Sigma), which were used for cDNA library construction using Illumina TruSeq RNA sample preparation kit (Illumina, Inc., San Diego, CA). The cDNA libraries were loaded on to a HiSeq Rapid flow cell, then paired‐end sequencing with 108 bp read length was performed on Illumina HiSeq 2500 (Illumina, Inc., San Diego, CA). RNA‐Seq reads were mapped using TopHat2 (Kim *et al*., 2013) onto a transcriptome reference database, and gene expression was quantified using Cufflinks2. Differential reads were determined by applying a parametric *t*‐test, with significance achieved at *P* < 0.05 and log_2_ > 1 in either direction. The expression value for each transcript was stated as RPKM, and three biological replicates were prepared for this experiment.

### qRT‐PCR analysis

We selected 10 differentially expressed genes from RNA‐sequencing for quantitative real‐time PCR analysis. The primers for these genes were designed using the Primer Premire5 software (PREMIER Biosoft, CA; Table S3). The reverse transcription was carried out using 1 μg of DNase‐treated total RNAs and the SuperScript^™^ VILO^™^ Master Mix (Invitrogen, Carlsbad, CA). The cDNA templates were amplified with Applied Biosystems 7500 Real‐Time PCR detection system and the SYBR Green JumStart^™^ Taq ReadyMix^™^ (Sigma, St. Louis, MO). The cotton *UBQ7* was used as the internal reference gene, and the relative transcript level of each gene was calculated using the 2^−ΔΔCt^ method (Livak and Schmittgen, 2001).

### Statistical analysis

Statistical analyses were performed in the R environment. Tukey's method was used for pairwise comparison among more than two groups of plants (WT, SNT, A, O and OA plants) at the significant level of *α* = 0.05.

## Conflict of interest

The authors declare that they have no competing interests.

## Author contributions

N.E., G.S., P.P. and H.Z. conceived and designed the experiments; N.E., Y.C., F.T., X.Z., J.S. and N.M. performed the experiments; N.E. and P.P. analysed the sequencing data; N.E., G.R., E.H., D.J., P.P. and H.Z. analysed the data and wrote the manuscript.

## Supporting information


**Figure S1** Performance of control and *OsSIZ1/AVP1* co‐overexpressing cotton plants under normal growth condition as well as under combined drought and salt stresses in greenhouse.
**Figure S2** Performance of control and *OsSIZ1/AVP1* co‐overexpressing cotton plants under normal growth condition as well as under combined drought and heat stresses in growth chamber.
**Figure S3** Performance of control and *OsSIZ1/AVP1* co‐overexpressing cotton plants in the field.
**Figure S4** Performance of control and *OsSIZ1/AVP1* co‐overexpressing cotton plants in the field.
**Figure S5** Analysis of fibre quality of control and *OsSIZ1/AVP1* co‐overexpressing cotton plants under rain‐fed conditions in the field in 2016.
**Table S1** List of primers used to amplify cDNAs for RNA and DNA blot analyses.
**Table S2** Rainfall and temperature information for Lubbock, Texas in 2016 and 2017.
**Table S3** List of primers used in quantitative real‐time PCR analyses.
