# Supplementary material for: Towards doubling fibre yield for cotton in the semiarid agricultural area by increasing tolerance to drought, heat and salinity simultaneously
Source: Plant Biotechnol J. 2020 Sep 29;19(3):462–76. doi: 10.1111/pbi.13476 (PMC7955890; doi:10.1111/pbi.13476)
Supplement: Supplementary file 1 — Figure S1 Performance of control and OsSIZ1/AVP1 co‐overexpressing cotton plants under normal growth condition as well as under combined drought and salt stresses in greenhouse. Figure S2 Performance of control and OsSIZ1/AVP1 co‐overexpressing cotton plants under normal growth condition as well as under combined drought and heat stresses in growth chamber. Figure S3 Performance of control and OsSIZ1/AVP1 co‐overexpressing cotton plants in the field. Figure S4 Performance of control and OsSIZ1/AVP1 co‐overexpressing cotton plants in the field. Figure S5 Analysis of fibre quality of control and OsSIZ1/AVP1 co‐overexpressing cotton plants under rain‐fed conditions in the field in 2016. Table S1 List of primers used to amplify cDNAs for RNA and DNA blot analyses. Table S2 Rainfall and temperature information for Lubbock, Texas in 2016 and 2017. Table S3 List of primers used in quantitative real‐time PCR analyses. [file PBI-19-462-s001.docx]

**Supplementary Figures**

**
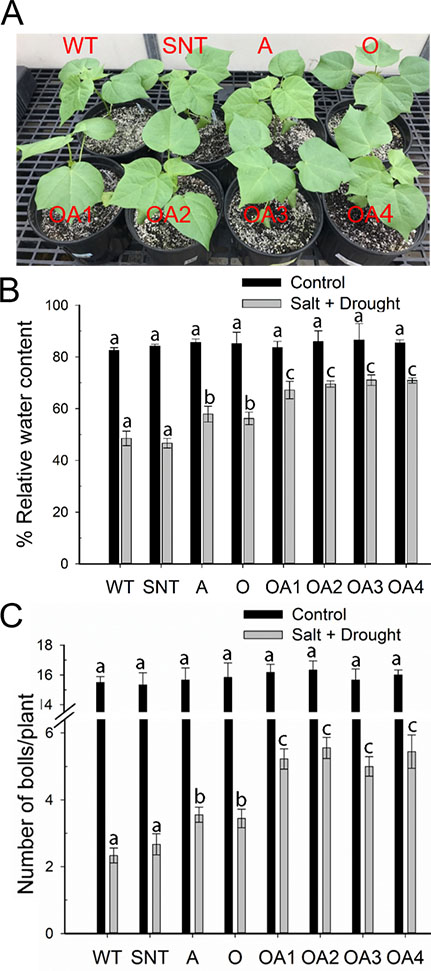
**

**Supp. Fig. 1.** Performance of control and *OsSIZ1/AVP1* co-overexpressing cotton plants under normal growth condition as well as under combined drought and salt stresses in greenhouse**. A.** Phenotype of cotton plants before drought and salt treatment. **B.** Relative water content of cotton plants under normal growth condition (black bars) and combined salt and drought stresses (grey bars). Data are the means ± SE (n = 5). **C.** Number of bolls produced per plant of cotton plants grown under normal growth condition (black bars) and combined drought and salt condition (grey bars). Data are the means ± SE (n = 9). WT, wild-type plant; SNT, segregated non-transgenic plant; A, *AVP1*-overexpressing plant; O, *OsSIZ1*-overexpressing plant; OA1 to OA4, four independent *OsSIZ1/AVP1* co-overexpressing plants. Samples denoted by different letters are significantly different (*P* < 0.05, Tukey correction).


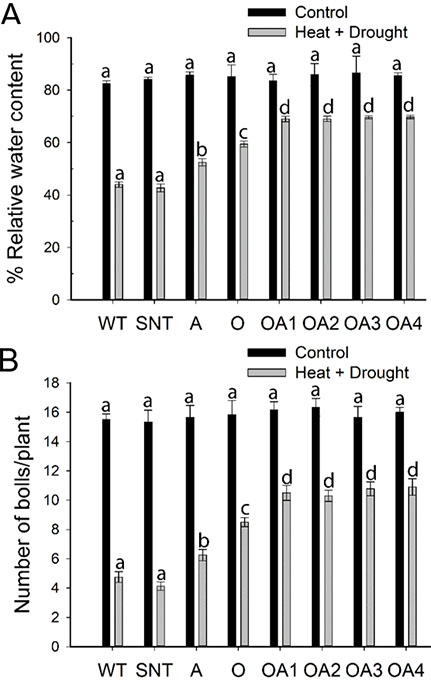


**Supp. Fig. 2.** Performance of control and *OsSIZ1/AVP1* co-overexpressing cotton plants under normal growth condition as well as under combined drought and heat stresses in growth chamber**. A.** Relative water content of cotton plants under normal growth condition (black bars) and under combined drought and heat stresses (grey bars). Data are the means ± SE (n = 5). **B.** Number of bolls produced per plant of cotton plants under normal growth condition (black bars) and combined drought and heat stresses (grey bars). Data are the means ± SE (n = 6). WT, wild-type plant; SNT, segregated non-transgenic plant; A, *AVP1*-overexpressing plant; O, *OsSIZ1*-overexpressing plant; OA1 to OA4, four independent *OsSIZ1/AVP1* co-overexpressing plants. Samples denoted by different letters are significantly different (*P* < 0.05, Tukey correction).

**
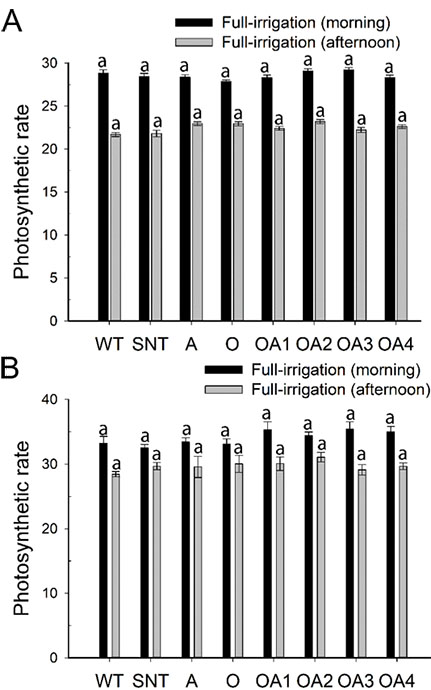
**

**Supp. Fig. 3.** Performance of control and *OsSIZ1/AVP1* co-overexpressing cotton plants in the field. **A.** Photosynthetic rates of control and *OsSIZ1/AVP1* co-overexpressing cotton plants under full-irrigation condition in 2016 in the morning (black bars) and in the afternoon (grey bars). **B.** Photosynthetic rates of control and *OsSIZ1/AVP1* co-overexpressing cotton plants under full-irrigation condition in 2017 in the morning (black bars) and in the afternoon (grey bars). Data are the means ± SE (n = 6). WT, wild-type plant; A, *AVP1*-overexpressing plant; O, *OsSIZ1*-overexpressing plant; OA1 to OA4, four independent *OsSIZ1/AVP1* co-overexpressing plants. Samples denoted by different letters are significantly different (*P* < 0.05, Tukey correction).

**
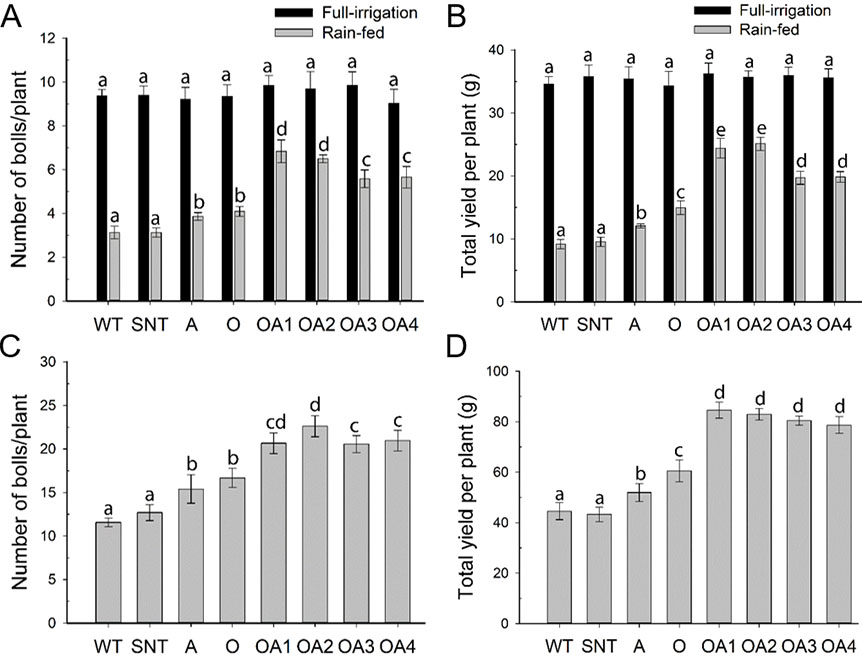
**

**Supp. Fig. 4.** Performance of control and *OsSIZ1/AVP1* co-overexpressing cotton plants in the field. **A.** Number of bolls produced per plant of control and *OsSIZ1/AVP1* co-overexpressing cotton plants in 2016 under full-irrigation (black bars) and under rain-fed (grey bars) conditions. **B.** Total seed fiber yield per plant of control and *OsSIZ1/AVP1* co-overexpressing cotton plants in 2016. **C.** Number of bolls produced per plant of control and *OsSIZ1/AVP1* co-overexpressing cotton plants under rain-fed conditions in 2017. **D.** Total seed fiber yield per plant of control and *OsSIZ1/AVP1* co-overexpressing cotton plants under rain-fed conditions in 2017. WT, wild-type plants; SNT, segregated non-transgenic plants; A, *AVP1*-overexpressing plants; O, *OsSIZ1*-overexpressing plants; OA1-OA4, four independent *OsSIZ1/AVP1* co-overexpressing plants. Samples denoted by different letters are significantly different (*P* < 0.05, Tukey correction).


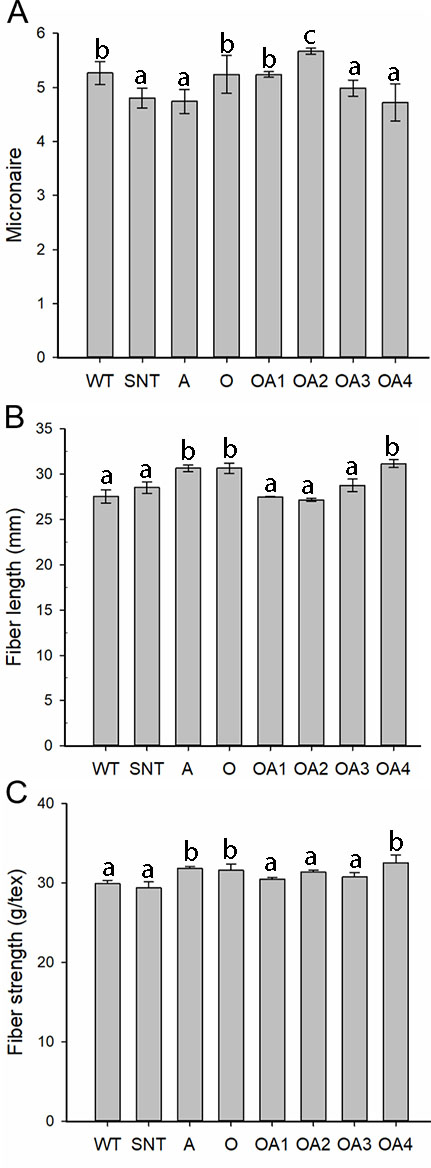


**Supp. Fig. 5.** Analysis of fiber quality of control and *OsSIZ1/AVP1* co-overexpressing cotton plants under rain-fed conditions in the field in 2016. Data are the means ± SE (n = 4). WT, wild-type plants; SNT, segregated non-transgenic plants; A, *AVP1*-overexpressing plants; O, *OsSIZ1*-overexpressing plants; OA1 to OA4, four independent *OsSIZ1/AVP1* co-overexpressing plants. Samples denoted by different letters are significantly different (*P* < 0.05, Tukey correction).

**Suplementary Tables**

**Supp. Table 1**. List of primers used to amplify cDNAs for RNA and DNA blot analyses.

*OsSIZ1* F: 5’-ATGGCGGACCTGGTTTCCAG-3’

R: 5’-ATAGTGACAGTGATTTGGAA-3’

*AVP1* F: 5’-ATGGGCGAGCTCGGTACC-3’

R: 5’-GAGAGACTGGTGATTTGCGGAC-3’

*Ubi7* F: 5’-CCTAGCCGCTGTACTTCTACTCCC-3’

R: 5’-GGACTCTACTCAATCCCCACCAG-3’

*NptII* F: 5’-GATTGAACAAGATGGATTGCACG-3’

R: 5’-CCCGATCATATTGTCGCTCAGG-3’

**Supp. Table 2**. Rainfall and temperature information for Lubbock, Texas in 2016 and 2017. (<https://www.usclimatedata.com/climate/lubbock/texas/united-states/ustx2745>).

**Month Average Precipitation (mm) Average High Temperature (°C)**

**2016 2017 2016 2017**

January 7.7 51.6 12.9 13.8

February 2.3 22.6 18.3 20

March 5 17 22.6 23.7

April 26 33.6 24.9 24.8

May 93.1 14.7 27.3 29.7

June 26.4 45.2 33 34.7

July 14.8 148.4 37.5 33.9

August 76.9 123.3 32.6 30.8

September 37.4 88 29 29.3

October 26.8 12.8 27.7 24.3

November 13.8 0.8 19.7 21.1

December 12.5 0 13.1 13.8

**Supp. Table 3**. List of primers used in quantitative real-time PCR analyses.

*RD22* F: 5’-GGCAAAGGTGGAGGAGTATCTGT-3’

R: 5’-TGGCAACTTCGTAAATGAAA-3’

*UBQ7* F: 5'-AGAGGTCGAGTCTTCGGACA-3’

R: 5'-GCTTGATCTTCTTGGGCTTG-3’

*PER53* F: 5’-GATATTCCCTAAGATCGTTCGCCT-3’

R: 5’-TCAACAGTAACGAAGCATCACAAC-3’

*HSP70* F: 5’-ACCCTAGCTGTTCTTTGTAGTAGC-3’

R: 5'-CCTTTACCGGCCATCACTATTTTC-3’

*RAB18* F: 5’-GAGGCTAAAGGAGAAGATACCAGG-3’

R: 5'-AGCTTCTCCTTGATCTTGTCCATC-3’

*HSP90* F: 5’-TGATAGCGGTATTGGCATGACTAA-3’

R: 5’-CCGAATTGTCCAATCATGCTAACA-3’

*HSFB2B* F: 5’-CGAAGCCTATTATATCACCGTCGA-3’

R: 5’ -CTGCACGTTCTCTTTCCTCAATTT-3’

*PIP2* F: 5’-TTGGCTCGTAAGGTATCATT-3’

R: 5’-CCTCCACCATACTTGTTGTA-3’

*SOD* F: 5’-TACTCCTGGGCCTCATGGCTT-3’

R: 5'-CTGCCACTCCATCAGCATTAG-3’

*CESA8* F: 5’-ACCCTGTAGATAAGGTCTCT-3’

R: 5'-TTTCCCGGCCAAGGAGTTCC-3’

*ARF1* F: 5’-ATCTGCGTGTTGGAGTGAGG-3’

R: 5’-CATGAGATGCAGTGGCAAGC-3’
